# Magnetic Tunnel Junction Based Long-Term Short-Term Stochastic Synapse for a Spiking Neural Network with On-Chip STDP Learning

**DOI:** 10.1038/srep29545

**Published:** 2016-07-13

**Authors:** Gopalakrishnan Srinivasan, Abhronil Sengupta, Kaushik Roy

**Affiliations:** 1 School of Electrical and Computer Engineering, Purdue University, West Lafayette, US

## Abstract

Spiking Neural Networks (SNNs) have emerged as a powerful neuromorphic computing paradigm to carry out classification and recognition tasks. Nevertheless, the general purpose computing platforms and the custom hardware architectures implemented using standard CMOS technology, have been unable to rival the power efficiency of the human brain. Hence, there is a need for novel nanoelectronic devices that can efficiently model the neurons and synapses constituting an SNN. In this work, we propose a heterostructure composed of a Magnetic Tunnel Junction (MTJ) and a heavy metal as a stochastic binary synapse. Synaptic plasticity is achieved by the stochastic switching of the MTJ conductance states, based on the temporal correlation between the spiking activities of the interconnecting neurons. Additionally, we present a significance driven long-term short-term stochastic synapse comprising two unique binary synaptic elements, in order to improve the synaptic learning efficiency. We demonstrate the efficacy of the proposed synaptic configurations and the stochastic learning algorithm on an SNN trained to classify handwritten digits from the MNIST dataset, using a device to system-level simulation framework. The power efficiency of the proposed neuromorphic system stems from the ultra-low programming energy of the spintronic synapses.

The advancements in computational neuroscience and cortical brain simulations have lead to tremendous progress in the development of complex brain-inspired computing systems over the past few years. Spiking Neural Networks constitute an important class of cognitive computing paradigms, and aim to mimic the computational efficiency of the human brain. The efficacy of SNNs in unsupervised pattern recognition has recently been demonstrated using a widely used handwritten digit recognition dataset^1^. However, the inability of the general-purpose computing platforms to harness the massive parallelism offered by SNNs, has resulted in the active exploration of energy efficient hardware implementations. Furthermore, the classical von-Neumann computing model, wherein the memory and processing cores are decoupled, are in stark contrast to the organization of the human brain. Hence, large-scale SNNs implemented on supercomputing clusters are extremely power hungry.

Significant efforts have been expended towards the development of custom hardware architectures for efficient realization of SNNs. The *SpiNNaker*^2^ and *IBM TrueNorth*^3^ are two such neuromorphic computing platforms, which were implemented using digital CMOS VLSI circuits. Such hardware implementations are still acutely power inefficient in comparison to the human brain. This can be attributed to the fundamental mismatch between the analog/digital CMOS circuits and the biological entities, namely the neurons and the synapses that are being modeled. However, we note that the power consumption of the CMOS neurons could be lowered by operating them in the sub-threshold region. On the other hand, synapses designed using 6T SRAM bitcells need to be operated at the nominal supply voltage so as to avoid memory failures in scaled technology nodes. This could be a potential bottleneck to minimizing the power consumption of such large-scale neuromorphic systems. This has lead to renewed interest in the exploration of novel post-CMOS technologies to emulate the biological neurons and synapses.

Emerging nonvolatile memory technologies including phase-change memories^4^, memristors^5^, and multilayer spintronic devices^6^,^7^ have been demonstrated to realize the synapses in a power efficient manner. We note that synapses in typical SNNs have traditionally been modeled as multilevel conductance as illustrated in Fig. 1. Interestingly, it has been observed that the signal propagation in a chemical synapse is regulated by the release of neurotransmitters, which is a stochastic process^8^. Additionally, multilevel memristive synaptic devices suffer from limited programming resolution in scaled technology nodes^9^. This has resulted in the exploration of alternative models of computation based on stochastic binary synapses. Nonvolatile resistive memories, for instance, the Spin Transfer Torque MTJ (STT-MTJ)^10^ and Conductive Bridge Memory (CBRAM)^11^, have been proposed to mimic a stochastic one-bit synapse. However, the algorithm used to effectuate plasticity in these devices is a simplified version of the biological learning rule^12^.

In this work, we present an MTJ-heavy metal (HM) heterostructure as a stochastic binary synapse. In the spike transmission mode of operation, the voltage spike produced by a pre-neuron is modulated by the conductance of the MTJ, thereby generating a resultant post-synaptic current. During the learning phase, a charge current is passed through the HM layer, which injects a spin current in the MTJ as a consequence of spin-Hall effect being the dominant physical mechanism. The proposed device thus provides decoupled spike-transmission and programming paths, which is an essential requirement of a nanoelectronic synapse subjected to on-chip learning^7^. Synaptic plasticity is effectively accomplished by the stochastic switching nature of the MTJ in the presence of thermal noise.

The binary synapses require a probabilistic learning mechanism to be able to acquire memory. We propose a stochastic algorithm that is consistent with the commandments of Hebbian learning^13^, in order to effectuate plasticity. Spike Timing Dependent Plasticity (STDP) is a widely used algorithm to accomplish unsupervised learning in SNNs. The synaptic strength is modulated based on the temporal correlation between the spiking patterns of pre-neuron and post-neuron pairs. For instance, in an SNN that is used for pattern recognition; the synapses fanning out of the region of interest in the input image, increase their strength (potentiate), while the insignificant synapses decrease their conductance (depress). However, in a binary synapse, potentiation (depression) would result in the maximum (minimum) value of conductance. Learning is essentially achieved by stochastically switching the synapses between their high and low conductance states. In this regard, we present a stochastic STDP algorithm, wherein the probability of potentiation and depression is exponentially related to the degree of temporal correlation between the spiking activities of pre-neuron and post-neuron pairs.

We observe that the intrinsic stochasticity results in spurious synaptic potentiation and depression events. This can be alleviated by acutely suppressing the switching activity with increasing differences in the spike times of pre-neuron and post-neuron pairs. However, this could adversely impact the capability of synapses to learn a generic representation of the input patterns. We propose a significance driven long-term short-term (LT-ST) synaptic memory, comprising two unique binary synapses to further improve the learning efficiency. This is inspired by the long-term and short-term partitions constituting the human memory^14^. The LT synapse is strengthened for strongly correlated input patterns, where the connected post-neuron fires immediately following an input pre-neuronal spike. On the other hand, the ST synapse potentiates with a greater probability for larger spike time differences so as to learn the distinct features of a class of input patterns. It also forgets at a higher rate than the LT synapse, which is therefore attributed a greater significance. We developed a device to system-level simulation framework to evaluate the proposed synaptic configurations. Our simulations show that the LT-ST synapse offers a better classification performance than the one-bit synapse on an SNN used for handwritten digit recognition. The key contributions of our work are:

(1) We explore an MTJ-HM heterostructure with independent spike-transmission and programming paths as a stochastic binary synapse.

(2) We propose the stochastic STDP algorithm and its circuit-level implementation, to train binary synapses in accordance with the principles of Hebbian learning.

(3) We present the significance driven long-term short-term stochastic synapse in order to further improve the learning efficiency of the SNN.

## Spiking Neural Network: Fundamentals

### Computational Model of the Neurons and Synapses

**Figure 1:**
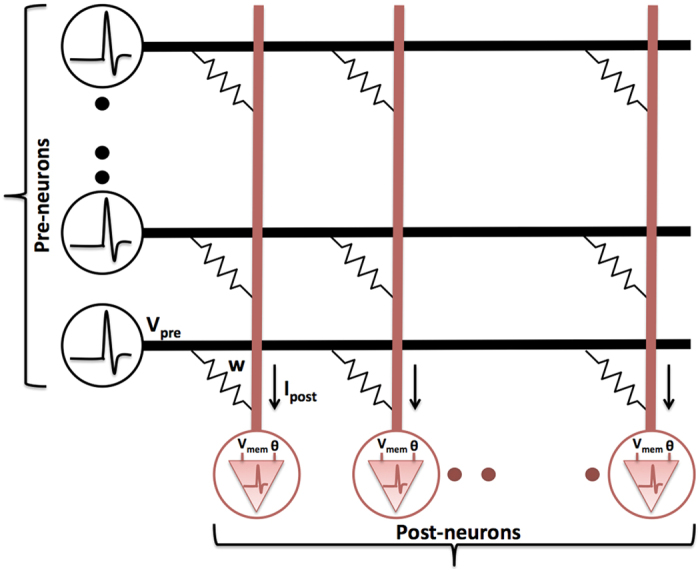
A typical SNN consisting of an array of pre-neurons and post-neurons interconnected by synapses that are modeled as multilevel conductance. The pre-synaptic voltage spike (*V*_*pre*_) is modulated by the synaptic conductance (*w*) to generate a resultant post-synaptic current (*I*_*post*_). The post-neuron integrates the current leading to an increase in its membrane potential (*V*_*mem*_), and spikes if the potential exceeds a certain threshold voltage (*θ*).

**Figure 2:**
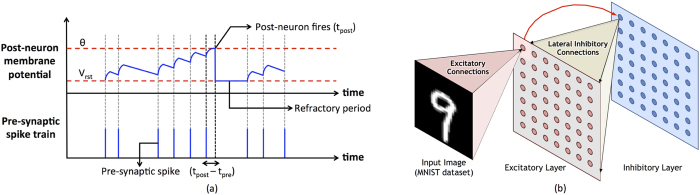
(**a**) The dynamics of the membrane potential of a post-neuron, which increases upon the arrival of an input pre-synaptic spike and decays subsequently. The post-neuron fires a spike if its membrane potential exceeds a definite threshold (*θ*). The potential is thereafter reset (*V*_*rst*_) and the neuron is restrained from spiking for a duration of time termed the refractory period. Synaptic potentiation depends on the period of time elapsed between a post-neuron spike and the most recent pre-synaptic spike (*t*_*post*_–*t*_*pre*_). (**b**) Hierarchical SNN topology for pattern recognition consisting of input, excitatory, and inhibitory layers. The input layer is fully connected to the excitatory neurons, which are connected to the corresponding inhibitory neurons in a one-to-one manner. Each of these neurons inhibits all the excitatory neurons except the one from which it received a forward connection.

The SNN topology, being utilized in this work for pattern recognition, consists of a couple of layers of spiking neurons interconnected by synapses as shown in Fig. 1. The neurons transmitting the spikes, for example, the vertical array of neurons in Fig. 1, are referred to as pre-neurons. Each pre-neuronal voltage spike is modulated by the synaptic conductance to produce a resultant post-synaptic current that is received by the post-neurons.


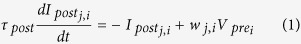


where 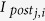 is the current received by the *j*^*th*^ post-neuron due to a voltage spike 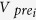 at the *i*^*th*^ pre-neuron, which are interconnected by a synapse of strength *w*_*j*,*i*_. The post-synaptic current increases momentarily at the instant of a pre-neuronal spike (*t*_*pre*_), and subsequently decays with the time constant *τ*_*post*_. The leaky integrate-and-fire (LIF) model^15^ is widely used to efficiently simulate the dynamics of a post-neuron and is illustrated in Fig. 2(a).


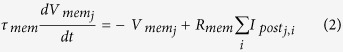


where 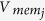 is the membrane potential of the *j*^*th*^ post-neuron, and *R*_*mem*_ is the membrane resistance. The post-neuron integrates the total post-synaptic current leading to an increase in its membrane potential, which eventually leaks with the time constant *τ*_*mem*_. A spike is fired if the membrane potential exceeds a definite threshold, and the time instant of its occurrence is denoted by *t*_*post*_. It is thereafter reset, and the neuron is prevented from spiking until a certain ensuing time interval designated as the refractory period. Synaptic plasticity depends on the interval of time elapsed between pairs of pre- and post-synaptic spikes as indicated in Fig. 2(a) for synaptic potentiation.

### SNN Topology for Pattern Recognition

A hierarchical SNN architecture^1^ as shown in Fig. 2(b), is used for pattern recognition. It consists of an input layer followed by the excitatory and inhibitory layers. The pixels in the image housing the definite patterns to be recognized constitute the input layer. They are converted to Poisson-distributed spike trains, wherein the rate of firing is proportional to the corresponding pixel intensities. The input neurons are fully connected to the neurons in the excitatory layer, each of which is trained to classify a specific input pattern. The excitatory neurons are then connected in a one-to-one fashion to the inhibitory neurons. Hence, an excitatory neuronal spike would cause the corresponding inhibitory neuron to fire. Every neuron in the inhibitory layer is connected back to all the excitatory neurons, saving the one from which it received a forward connection. Lateral inhibition thus promotes competitive learning, and effectively prevents all the excitatory neurons from learning similar patterns.

## Magnetic Tunnel Junction (MTJ)-Heavy Metal (HM) as a Stochastic Binary Synapse

A standalone MTJ can be used as a binary synapse^10^, owing to its inherent stochastic switching behavior. It consists of two ferromagnetic layers separated by a spacer layer (MgO). The magnetization of one of the layers can be switched by an input spin current, and is referred to as the *free layer* (FL). The other magnetic layer serves as the *reference* or *pinned layer* (PL), and is magnetostatically fixed. The MTJ exhibits two stable resistance states depending on the orientation of the FL and PL magnetizations. It is said to be in the low (high) resistance state, also referred to as the ‘Parallel’ (‘Anti-Parallel’) state, if the FL magnetization is in the same (opposite) direction relative to the PL.

Let us consider the MTJ switching dynamics when the spin current is generated by a charge current flowing through the HM layer, which is located underneath the MTJ structure. This results in a transverse spin current on the top and bottom surfaces of the HM due to spin-Hall effect, which results in switching of the MTJ (with in-plane magnetic anisotropy)^16^,^17^. The spin-Hall effect induced switching of the MTJ has been shown to be much more energy-efficient, since electrons flowing through the HM can transfer multiple units of angular momentum. This can be attributed to the repeated scattering of electrons at the interface of FL and HM.

**Figure 3:**
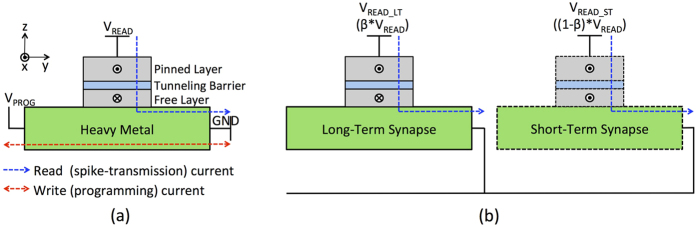
(**a**) Cross-sectional view of an MTJ-HM binary synapse. The read (spike-transmission) current flowing between the terminals *V*_*READ*_ and *GND* is modulated by the MTJ conductance, while the write (programming) current flowing between terminals *V*_*PROG*_ and *GND* stochastically switches the magnetization of the free layer. (**b**) A significance driven LT-ST stochastic synapse comprising two MTJ-HM devices. The LT synapse is driven by a relatively higher read voltage (*V*_*READ*_*LT*_ > *V*_*READ*_*ST*_; *β* > 0.5), thereby leading to a larger post-synaptic current.

The MTJ-HM multilayer structure gives rise to a three terminal binary synaptic device with decoupled write and read current paths as indicated in Fig. 3(a). Such a device configuration is indispensable in SNNs requiring on-chip learning, in order to resolve potential read–write conflicts^7^. While the write current flows through the HM underlayer, the read current flows through the MTJ and is modulated by its conductance. It is worth noting that the direction of write current through the HM underlayer determines the final resistance state of the MTJ. Furthermore, we propose a significance driven LT-ST stochastic synapse that is formed by two discrete MTJ-HM devices as illustrated in Fig. 3(b). The LT synaptic device carries a greater significance, and hence, is driven by a relatively higher read voltage so as to produce a larger post-synaptic current. Finally, we note that both the proposed synaptic configurations rely on the probabilistic switching nature of the MTJ in the presence of thermal noise at non-zero temperatures.

### Device Simulations

The magnetization dynamics of the FL of an MTJ at zero temperature can be obtained empirically, by solving the *Landau-Lifshitz-Gilbert-Slonczewski* (LLGS) equation^18^.


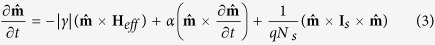


where 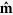 is the unit vector pointing in the direction of the FL magnetization, 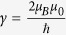 is the gyromagnetic ratio for an electron, **H**_*eff*_ is the effective magnetic field incorporating the shape anisotropy for an elliptical disk^19^, *α* is the Gilbert’s damping ratio, 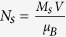 is the number of spins in the FL occupying a definite volume *V*, *M*_*s*_ is the saturation magnetization, *μ*_*B*_ is Bohr magneton, and *μ*_0_ is the magnetic permeability. The spin current, **I**_*s*_, is produced by a charge current injected into the HM underlayer.


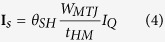


where *θ*_*SH*_ is the spin-Hall angle^16^, *W*_*MTJ*_ is the width of the MTJ, *t*_*HM*_ is the thickness of the HM, and *I*_*Q*_ is the amount of charge current flowing through the HM. It is worth noting that such a device can potentially achieve a spin injection efficiency of greater than 100%, since the spin polarization is not limited by the polarization of the PL.

**Figure 4:**
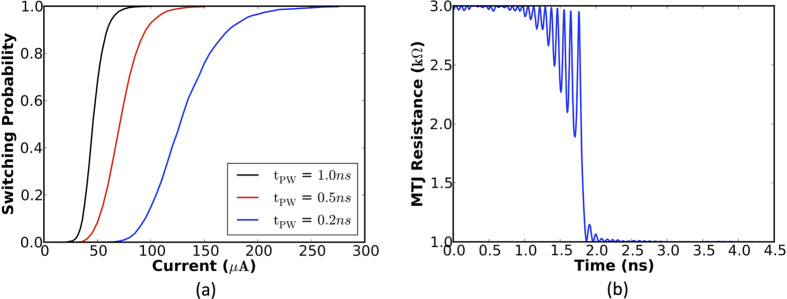
(**a**) The switching probability of the free layer magnetization of the MTJ due to a programming current flowing through the HM underlayer. (**b**) Change in the MTJ resistance upon the application of a 40 *μA* current pulse for a duration of 1 *ns*.

At non-zero temperatures, the magnetization characteristics of the MTJ are impacted by thermal noise, which is factored into the LLGS equation by augmenting **H**_*eff*_ with a thermal field, **H**_*thermal*_^20^.


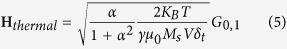


where *G*_0,1_ is a Gaussian random variable with zero mean and unit standard deviation, *K*_*B*_ is the Boltzmann constant, *T* is the temperature, and *δ*_*t*_ is the simulation time step. Hence, an MTJ exhibits stochastic switching dynamics in the presence of thermal noise. It can be deduced from Fig. 4(a) that the switching probability increases with the magnitude of the write current. Figure 4(b) illustrates the change in the MTJ resistance upon the application of a large enough write current. The device simulation parameters are listed in Table 1.

## Stochastic Synapse Configurations and Learning Methodology

### Exponential STDP for a Multilevel Synapse

In the SNN used for pattern recognition, the synapses interconnecting the input neurons (pre-neurons) to each excitatory neuron (post-neuron) need to be trained to encode a generic representation of a class of input patterns. According to the exponential STDP learning algorithm, the strength of a multilevel synapse is regulated depending on the difference in the spike times of pre-neuron and post-neuron pairs. The conductance is increased/potentiated if a pre-neuronal spike subsequently causes a post-neuron to fire. On the other hand, it is decreased/depressed if a pre-neuron fires following a post-neuronal spike, which indicates the absence of a causal relationship. The mathematical formulation of STDP is derived from the synaptic plasticity mechanism observed in the rat hippocampus^12^.

### Stochastic STDP for a Binary Synapse

We infer from the exponential STDP dynamics that the synapses encoding an input pattern are gradually strengthened, effectively resulting in a larger conductance towards the end of the training phase. On the contrary, the insignificant synapses are eventually forced to a low value of conductance. Intuitively, a binary synapse comprising high and low conductance states could be trained to learn specific patterns. However, a stochastic learning algorithm is required to implement plasticity, so as to prevent rapid instantaneous synaptic switching that could ultimately render the synapses memory-less.

**Figure 5:**
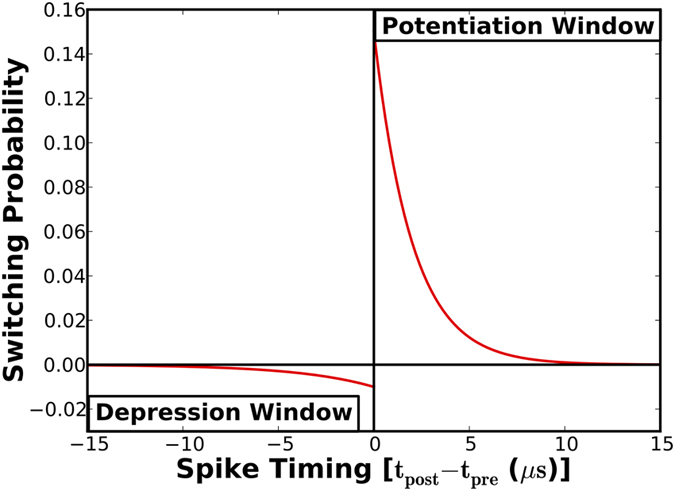
Stochastic STDP dynamics of a one-bit synapse, wherein the probability of potentiation and depression is exponentially related to the difference in the spike times of a post-neuron (*t*_*post*_) and pre-neuron (*t*_*pre*_).

In this regard, we present the stochastic STDP learning mechanism, wherein the synaptic switching probability is contingent on the temporal correlation between the spiking activities of the interconnecting neurons. If a pre-neuronal spike forces a post-neuron to fire, the corresponding synapse should potentiate probabilistically since it is positively correlated with the input pattern. On the contrary, if a pre-neuron fires following a post-neuronal spike, the specific synapse ought to depress conditionally since it is negatively correlated with the input pattern. We note that potentiation (depression) occurs in the positive (negative) timing window of the STDP algorithm. The probability of potentiation and depression is exponentially related to the difference in the spike times of pre-neuron and post-neuron pairs, as shown in Fig. 5. This is an advancement over prior research efforts^10^,^21^ that used a constant switching probability, thereby disregarding the spike-timing information.


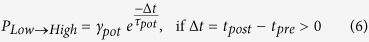



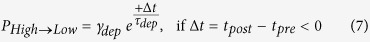


where *P*_*Low*→*High*_ and *P*_*High*→*Low*_ are the probability of potentiation and depression respectively. The learning efficacy is dependent both on the peak switching probability and the ratio of the time constants governing the rate of potentiation (*γ*_*pot*_, *τ*_*pot*_) and depression (*γ*_*dep*_, *τ*_*dep*_). It can be observed from Fig. 5 that the peak probability of potentiation is limited to 15%. This is because a larger switching probability causes the synapses to learn specific training patterns rather than a generic representation of a class of input patterns. Moreover, a larger depression probability could potentially cause them to forget features that are common to different input classes. Hence, the STDP parameters must be tuned to strike a good balance between classification performance and the number of training epochs required for achieving convergence. In the following sub-section, we present a two-bit stochastic synapse that is inspired by the neuroscience mechanisms in order to further improve the learning efficiency.

### Significance Driven Long-Term Short-Term Synapse

**Figure 6:**
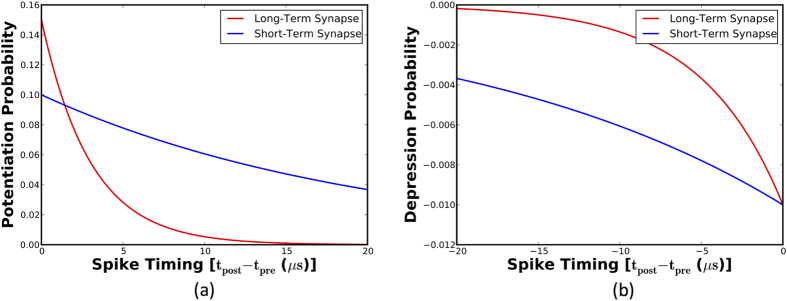
STDP dynamics of an LT-ST stochastic synapse. (**a**) The LT synapse has a sharper rate of potentiation that enables it to learn strongly correlated input patterns while the ST synapse potentiates with a greater probability for larger spike time differences to acquire moderately correlated features. (**b**) The LT synapse has a steeper rate of depression to reliably retain the acquired information while the ST synapse has a larger depression probability over the negative STDP timing window.

The long-term short-term (LT-ST) stochastic synapse is composed of two individual one-bit synaptic elements. The LT synapse has a sharper rate of potentiation as evidenced by Fig. 6(a), and therefore learns strongly correlated input patterns. Additionally, the steeper rate of depression enables it to retain the acquired information in a reliable manner. However, the sharper STDP dynamics could restrict its capability to encode a comprehensive representation of a class of input patterns. On the other hand, the ST synapse potentiates with a higher probability for larger differences in the spike times of pre-neuron and post-neuron pairs. It is therefore more likely to potentiate for various distinct features of a specific pattern that could have a moderate correlation. Furthermore, the increased probability of depression over the negative STDP timing window causes it to forget at a higher rate relative to the LT synapse. This enables it to counterbalance the impact of unintended synaptic potentiations caused by the augmented probability of switching in the positive STDP timing window.

In addition to the distinctive STDP dynamics, the proposed configuration attributes varying significance to the constituent synaptic elements to achieve improved learning efficiency. We note that the resultant post-synaptic current is a linear combination of definite contributions from the LT and ST synaptic elements, both of which are driven at a voltage lower than that used to read a unit one-bit synapse as illustrated in Fig. 3(b). The total current generated by a fully potentiated LT-ST synapse is equal to that produced by a unit one-bit synapse. The LT synapse is assigned a relatively greater significance since it learns strongly correlated input patterns. Hence a spike propagating through the former contributes to a larger post-synaptic current. We observe that the probabilistic nature of switching could cause a loosely correlated LT or ST synapse to potentiate. The resultant post-synaptic current in such a scenario is only a fraction of the current that would be generated as a result of an undesirable potentiation of a unit one-bit synapse. In the worst-case scenario, unintended potentiation of both the LT and ST synaptic elements would result in a current that is comparable to that generated by a one-bit synapse. Hence, the LT-ST synaptic configuration is tolerant to spurious switching events relative to a one-bit synapse with an increased rate of potentiation. The proposed configuration can be tuned to encode a better representation at a faster convergence rate by striking a balance between the switching probabilities and the relative significance of the constituent synaptic elements.

## Spiking Neuromorphic System Architecture

### Stochastic STDP Implementation

**Figure 7:**
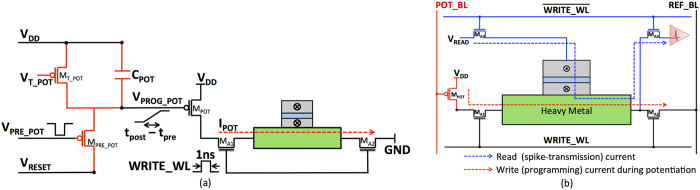
(**a**) Implementation of the stochastic STDP algorithm. The control signal *V*_*PRE*_*POT*_ resets the node voltage *V*_*PROG*_*POT*_ following an input pre-neuron spike. Once *M*_*PRE*_*POT*_ turns off, *V*_*PROG*_*POT*_ increases linearly at a rate determined by *C*_*POT*_ and the bias voltage *V*_*T*_*POT*_. The access transistors *M*_*A*1_–*M*_*A*2_ are enabled by the control signal WRITE_WL, which is asserted high at an appropriate instant following a spike fired by the connected post-neuron. Finally, *V*_*PROG*_*POT*_ that is proportional to the elapsed time period between a pair of pre-and post-neuron spikes (*t*_*post*_–*t*_*pre*_) is sampled by *M*_*POT*_ to generate the required write current *I*_*POT*_. A similar discussion is valid for the depression circuit, wherein the programming current is passed in the opposite direction. (**b**) Bitcell configuration of an MTJ-HM binary synapse. The bitline POT_BL drives the potentiation circuit path, which is conditionally enabled by the write wordline (WRITE_WL). The reference bitline (REF_BL) is driven to ground. The spike-transmission path is driven by the pre-neuronal voltage spike (*V*_*READ*_), and is active whenever the WRITE_WL is driven low.

The stochastic learning algorithm specifies the probability of potentiation and depression based on the spike-timing information. An appropriate amount of current needs to be passed through the HM layer to switch the MTJ conductance states with the determined probability. It can be inferred from the stochastic STDP dynamics (Fig. 5) and the MTJ switching characteristics for a programming pulse width of 1 *ns* (Fig. 4(a)) that the write current decreases linearly with the difference in spike times of pre-neuron and post-neuron pairs. Figure 7(a) illustrates a CMOS implementation of the stochastic STDP algorithm during the positive timing window. The circuit was originally presented to operate as a reset-and-discharge synapse^22^. We use it to implement the proposed stochastic learning algorithm in the MTJ-HM device as described below.

An active low control signal *V*_*PRE*_*POT*_ turns on the transistor *M*_*PRE*_*POT*_, which acts as a switch and resets the node voltage *V*_*PROG*_*POT*_ at a suitable instant following an input pre-neuron spike (*t*_*pre*_). Once *M*_*PRE*_*POT*_ turns off, the transistor *M*_*T*_*POT*_ operating in sub-threshold saturation charges the *V*_*PROG*_*POT*_ node of the capacitor *C*_*POT*_. The voltage *V*_*PROG*_*POT*_ increases linearly at a rate determined by the capacitance *C*_*POT*_ and the bias voltage *V*_*T*_*POT*_. The access transistors *M*_*A*1_–*M*_*A*2_ are enabled by the control signal WRITE_WL at an appropriate instant following the excitatory post-neuron spike (*t*_*post*_). The programming voltage *V*_*PROG*_*POT*_ is thus proportional to the interval of time elapsed between the assertion of the control signals *V*_*PRE*_*POT*_ and WRITE_WL, which are synchronous to the pre-neuron (*t*_*pre*_) and post-neuron (*t*_*post*_) spike times respectively. This drives the transistor *M*_*POT*_ operating in the saturation region that is sized to generate the required amount of write current *I*_*POT*_.

A similar circuit using an NMOS transistor biased in saturation generates the write current required to achieve synaptic depression. The depression circuit is activated whenever a pre-neuron spikes while the corresponding potentiation circuit is enabled with an added delay of the negative STDP timing window, using the control signal *V*_*PRE*_*POT*_. The respective programming currents are sampled sequentially whenever a post-neuron spikes, however with an additional delay corresponding to the duration of the negative STDP timing window. It is important to note that the write current during depression flows in a direction opposite to that during potentiation. The learning circuit thus accounts for both the negative and positive timing windows involved in the STDP algorithm.

### Spintronic Synapse: Bitcell Configuration

In this sub-section, we discuss the bitcell configuration of the MTJ-HM spintronic synapse. The proposed three-terminal synaptic device has two primary modes of operation, namely, the read (spike-transmission) mode and the programming mode as shown in Fig. 7(b). The programming mode further involves the potentiation and depression phases. The access transistors *M*_*A*1_–*M*_*A*4_ activate the circuit paths that are pertinent to the various modes of operation.

During the read mode of operation, the write wordline (WRITE_WL) is driven low, thereby turning on the access transistors *M*_*A*3_–*M*_*A*4_. The input pre-neuronal voltage spike (*V*_*READ*_) propagates through the MTJ and gets modulated by its conductance, effectively generating a post-synaptic current. The WRITE_WL is asserted high once the post-neuron spikes with a delay equal to the negative STDP timing window, which enables the programming path. The voltage *V*_*PROG*_*POT*_ is applied to the potentiation bitline (POT_BL), while the reference bitline (REF_BL) is driven to ground. The transistor *M*_*POT*_ samples *V*_*PROG*_*POT*_ and generates the necessary programming current. It needs to be noted that a complementary circuit using an NMOS transistor is used to implement synaptic depression. The resultant write current flows through the HM layer in a direction opposite to that during potentiation. The WRITE_WL is successively asserted high at suitable instants subsequent to a post-neuron spike in order to accomplish both synaptic potentiation and depression. This is a feasible approach since the spike times are in in the order of *μs*, while the write pulse width spans a few *ns*.

The primary advantage of the presented MTJ-HM synapse is that it is a three-terminal device with decoupled read–write paths, which enables the read and programming circuit paths to be optimized independently for energy efficient operation. On the other hand, two-terminal synaptic devices such as memristors share the read–write path, which results in conflicting optimization requirements for the respective operations. The synaptic bitcell illustrated in Fig. 7(b) incurs comparatively less area penalty than a CMOS based synaptic circuit implemented using the 6T SRAM technology, which requires 24 transistors for a precision of 4 bits. Furthermore, a binary synapse implemented using 6T SRAM would require additional overheads for incorporating the stochastic learning algorithm.

Each synaptic unit (bitcell) consists of an MTJ-HM device, two programming transistors for generating the write current during synaptic potentiation and depression, and four access transistors for eliminating the sneak paths. We note that transistor gating is widely used to address the issue of sneak paths in crossbar architectures irrespective of the technology used to realize the synapses. Alternative architectures utilizing time division multiplexing^5^ have been proposed, which preclude the requirement of gating transistors at every cross point. Such a scheme operates the entire crossbar array in successive read and write cycles. This leads to an increase in the energy consumption due to redundant read and write operations that are carried out even in the absence of spiking events. Hence, our design choice of using access transistors to eliminate the sneak paths provides a reduction in the system energy consumption.

It is important to note that the STDP learning circuit illustrated for synaptic potentiation consisting of two transistors and a capacitor (*M*_*PRE*_*POT*_, *M*_*T*_*POT*_, and *C*_*POT*_ in Fig. 7(a)) is not contained within a synaptic unit. It is shared by the synapses connecting an input neuron to all the excitatory neurons as will be explained in the sub-section on spintronic crossbar architecture. We note that neuromorphic architectures supporting on-chip learning require an analog/digital CMOS interfacing circuit to incorporate the learning algorithm^23^,^24^.

### Circuit Simulations

**Figure 8:**
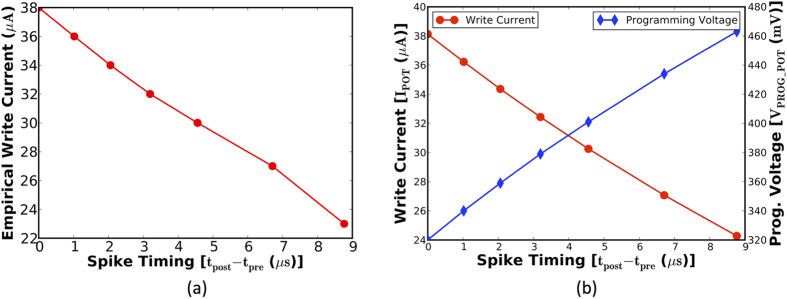
(**a**) Write current required to achieve stochastic potentiation of an MTJ-HM spintronic synapse. The corresponding stochastic STDP dynamics are characterized by a peak switching probability (*γ*_*pot*_) of 0.15 and a potentiation time constant (*τ*_*pot*_) of 2 *μs*. (**b**) SPICE simulation of the STDP learning circuit, wherein the programming voltage is proportional to the difference in the spike times of pre-neuron and post-neuron pairs. This drives a PMOS operating in saturation to produce a linearly decreasing write current.

The CMOS implementation of the stochastic STDP algorithm for a one-bit synapse was verified by carrying out SPICE simulations in 45 *nm* technology node using the parameters listed in Table 2. Figure 8(a) illustrates the linear variation in the write current required to accomplish stochastic synaptic potentiation, which conforms to the STDP dynamics depicted in Fig. 5 and the MTJ switching characteristics shown in Fig. 4(a) for a programming pulse width of 1 *ns*. The rate of change of write current and programming voltage in the positive (negative) STDP timing window are strongly dependent on the time constants governing the rate of potentiation (depression), aside from the MTJ switching dynamics.

The precise operation of the STDP learning circuit is validated by Fig. 8(b). The programming voltage increases linearly with the difference in the spike times of pre-neuron and post-neuron pairs. The rate of increase of *V*_*PROG*_*POT*_ is modulated by appropriately sizing *C*_*POT*_ and applying a suitable bias (*V*_*T*_*POT*_) to the transistor *M*_*T*_*POT*_, and the respective values are listed in Table 2 for the proposed synaptic configurations. It is worth noting that the circuit parameters for an LT synapse are different than those for an ST synapse, since the individual synaptic elements possess unique STDP characteristics. The voltage *V*_*PROG*_*POT*_ directly feeds the gate of the transistor *M*_*POT*_ operating in saturation, which is sized to produce the required write current. The maximum value of the write current flowing through the HM layer is 38 *μA* for a duration of 1 *ns*. The learning circuit is operated at 1 *V*. The maximum energy consumed in programming the MTJ-HM synapse is roughly 38 *fJ*. This is energy efficient in comparison to a resistive RAM (RRAM) based synaptic element that was reported to consume 290 *fJ* per programming event^23^. We analyze the average power consumed by arrays of MTJ-HM synapses constituting an SNN trained for digit recognition in the results section.

### Spintronic Crossbar Architecture

**Figure 9:**
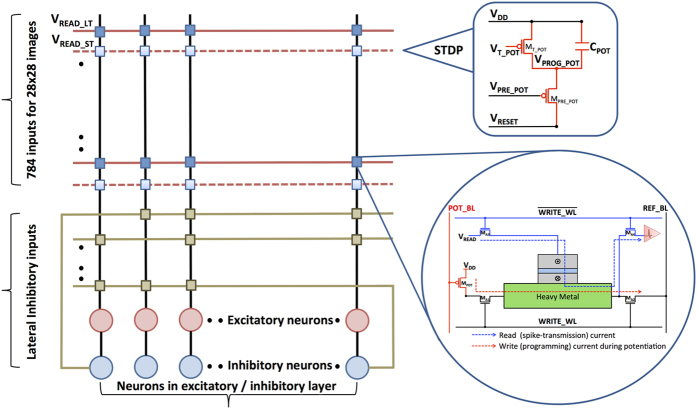
Architecture of an SNN composed of excitatory and inhibitory neurons, and arrays of LT-ST stochastic synapses (Bitcell shown in Fig. 7(b)). The LT synapse is operated at a higher voltage (*V*_*READ*_*LT*_) than the ST synapse (*V*_*READ*_*ST*_). The STDP learning circuit (Fig. 7(a)) that is illustrated here is shared by the horizontal array of synapses. The parameters of the learning circuit for the LT and ST synaptic arrays are mentioned in Table 2.

We present a crossbar architecture for an SNN consisting of excitatory and inhibitory neurons, and stochastic MTJ-HM binary synapses as shown in Fig. 9. We note that the LT-ST synapses are solely utilized for the connections between the input and excitatory neurons, since these are the only ones subjected to on-chip learning. During the read phase, each input pre-neuron spike propagates through the horizontal arrays of both LT and ST synapses. However, the relatively significant LT synapses generate a larger post-synaptic current since they are driven at a higher read voltage. Furthermore, a negative voltage spike drives the lateral inhibitory voltage lines so that the corresponding currents are subtracted. It is important to note that the STDP learning circuit illustrated for synaptic potentiation is shared by the synapses connecting an input neuron to all the excitatory neurons (horizontal array of synapses). The programming voltage *V*_*PROG*___*POT*_, which drives the potentiation bitline (POT_BL) in the corresponding row of synapses, is effectively reset at an appropriate instant following a pre-neuron spike. The write wordline (WRITE_WL) of each synaptic bitcell is activated at a suitable time instant subsequent to an excitatory post-neuron spike in order to achieve stochastic potentiation. It is worth mentioning that the synapses connecting an excitatory neuron to all the input neurons (vertical array of synapses) are programmed during the write phase, and hence share the respective write wordline. The superior performance of the crossbar organization can be attributed to the localized arrangement of the neurons and synaptic memories.

## Results and Discussion

### Simulation Methodology

We developed a complete device-circuit-functional simulation framework to evaluate the proposed synaptic configurations. Device simulations were initially carried out to obtain the switching characteristics of the MTJ-HM synaptic memory. As mentioned earlier, the stochastic STDP dynamics, i.e., the variation in the switching probability with spike-timing information, shown in Figs 5 and 6 were determined from system-level simulations. The respective STDP parameters were optimized to achieve the best trade-off between classification performance and the number of training epochs. These results were in turn used to establish the relationship between the synaptic programming current and neuronal spike times. This was central to designing the stochastic STDP learning circuit, which was verified using SPICE simulations in 45 *nm* technology node.

The one-bit and LT-ST stochastic synapses were simulated on a hierarchical SNN used to classify handwritten digits from the MNIST^25^ dataset. The stochastic STDP algorithm was implemented in BRIAN^26^, which is an open source large-scale SNN simulator. The network topology and the associated connectivity information were programmed in the simulator, which is equipped with a parameterized functional model of the leaky integrate-and-fire (LIF) neuron. The time instants of pre-neuron and post-neuron spikes were recorded to estimate the corresponding synaptic switching probability based on the stochastic STDP dynamics. The synapses were then conditionally depressed (potentiated) at the instant of a pre-neuron (post-neuron) spike to account for the negative (positive) timing window of the STDP algorithm. The efficacy of the algorithm was found to be dependent on the following system parameters that needed to be tuned in a holistic manner for efficient stochastic learning.

(1) The rate of spiking and leak conductance parameters of the excitatory neurons.

(2) The rate of spiking and leak conductance parameters of the inhibitory neurons. The lateral inhibition needed to be stronger during the learning phase to prevent the excitatory neurons from spiking for multiple input patterns.

(3) Stochastic STDP parameters, namely, the peak switching probability, and the rate of potentiation and depression.

The programming energy expended during the training phase is obtained by summing up the contributions from the STDP learning circuit that modulates the programming voltage based on spike timing and the synaptic bitcells that sample the voltage to generate the required write current through the MTJ-HM device. The energy consumption of the learning circuit shown in Fig. 7(a) for synaptic potentiation is estimated by integrating the energy needed to charge the *V*_*PROG*_*POT*_ node over the period of time elapsed between successive pre-synaptic spikes. It is important to note that the programming voltage *V*_*PROG*_*POT*_ is reset subsequent to the arrival of a pre-synaptic spike and increases until the arrival of the following spike.


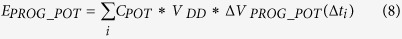


The parameters *C*_*POT*_ and *V*_*DD*_ are listed in Table 2 for the proposed synaptic configurations. The quantity Δ*V*_*PROG*_*POT*_(Δ*t*_*i*_) is the change in the voltage across *C*_*POT*_ over the time interval (Δ*t*_*i*_) between a pair of successive pre-synaptic spikes, and is computed based on the rate of increase of *V*_*PROG*_*POT*_ estimated from the circuit simulations (Fig. 8(b)). A similar methodology is used to estimate the energy consumption of the voltage modulation circuit for synaptic depression. The programming energy of the synaptic bitcell shown in Fig. 7(b) is obtained by integrating the energy consumed in passing the required amount of write current through the MTJ-HM device for a certain duration of time per synaptic update. Each bitcell additionally needs a WRITE_WL inversion circuit to decouple the read and write paths, and its energy consumption is added per synaptic update to obtain the total bitcell programming energy.


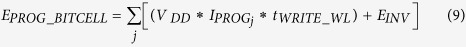


where 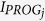 is the programming current during *j*^*th*^ synaptic update that is determined based on the spike timing information recorded during the functional simulation, *t*_*WRITE*_*WL*_ is chosen to be 1 *ns*, and *E*_*INV*_ is the energy consumed by the WRITE_WL inversion circuit, which is estimated to be 1 *fJ* per synaptic update for a 1 *fF* inverter load.

Once the training is complete with tuned system parameters, each excitatory neuron is assigned to a specific output class (label) based on the digit pattern for which it spiked the most during the learning phase. Digit recognition is then carried out by analyzing the spiking activity of different groups of neurons in the network, each of which belongs to a definite output category. Each input (test) image is predicted to belong to the output class represented by the group of neurons with the highest average spike count over the course of the simulation period. The classification is accurate if the actual label of a test image matches with that predicted by the network of spiking neurons. This is a commonly used approach to evaluate the classification performance of single layered SNNs^1^, and is illustrated with the following example. Consider that a test image, for instance ‘5’, is fed to the input neurons over a period of time. This could potentially cause several output neurons in the network to fire. The input image is correctly classified if the group of neurons that had learned to spike for different representations of ‘5’ during training, fired the maximum number of spikes. The classification accuracy is defined as the ratio of the number of images correctly classified by the trained network to the total number of test images. We used two thousand image samples from the MNIST testing dataset to report the classification performance. Note that the classification accuracy results were averaged across five presentations of the image dataset to account for the randomness involved in converting the pixel intensities to Poisson-distributed spike trains. Furthermore, the parameters governing the neuronal dynamics were kept identical to perform a fair comparison between the proposed synaptic configurations.

### Stochastic One-Bit Synapse

**Figure 10:**
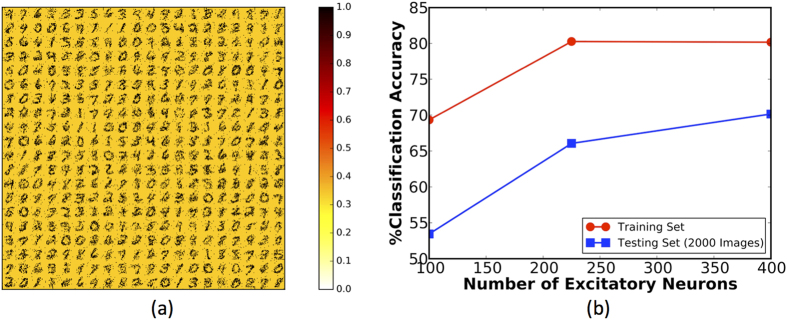
(**a**) Digit representations learned by arrays of stochastic one-bit synapses connecting the input (28 × 28 pixels) to each of the 400 excitatory neurons. The ratio of the minimum to maximum synaptic conductance is considered to be 1:3. (**b**) Classification accuracy of the SNN versus the number of excitatory neurons for both the testing and training dataset, with the impact of lateral inhibition reduced during the evaluation phase.

Figure 10(a) shows the final conductance states of the synapses connecting the input neurons to each of the 400 excitatory neurons. The synapses that were randomly initialized eventually learned to encode a generic representation of a class of input patterns towards the end of the training phase. This is a testament to the efficacy of the stochastic STDP learning algorithm. Figure 10(b) further shows that the classification accuracy can be improved by increasing the number of excitatory neurons. Nevertheless, the undesirable synaptic potentiations were found to have a detrimental impact on the classification performance of the SNN. In the following sub-section, we demonstrate a two-bit stochastic synapse inspired by the neuroscience mechanisms that further improves the efficiency of synaptic learning.

### Significance Driven LT-ST Stochastic Synapse

**Figure 11:**
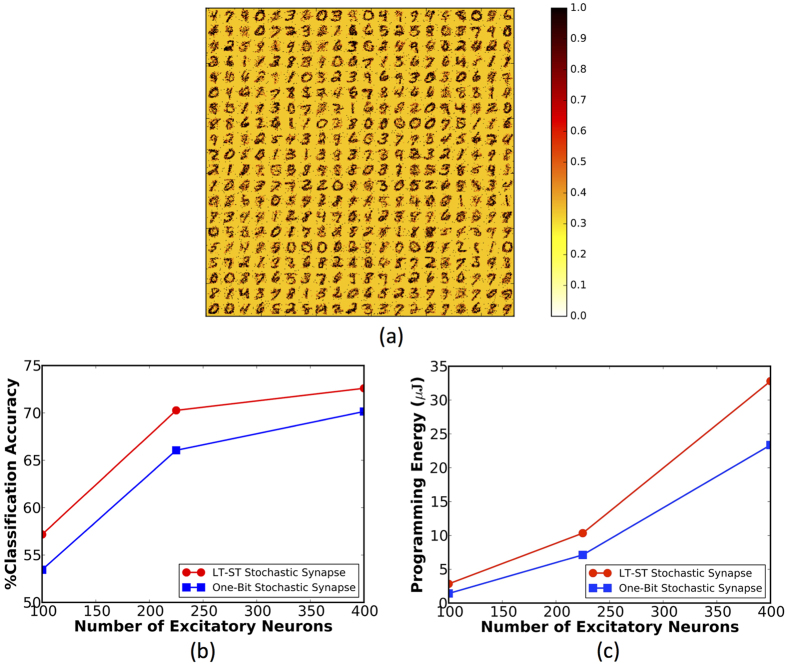
(**a**) Digit representations learned by arrays of LT-ST synapses connecting the input (28 × 28 pixels) to each of the 400 excitatory neurons. The significant LT synapses contribute to 80% of the total post-synaptic current. (**b**) Classification accuracy of the SNN versus the number of excitatory neurons for both the proposed synaptic configurations. (**c**) Programming energy consumption versus the number of excitatory neurons for both the proposed synaptic configurations.

This configuration offers improved learning capabilities as can be observed from Fig. 11(a), which demonstrates the patterns encoded by arrays of LT-ST synapses. The distinctive STDP dynamics along with the higher significance awarded to the LT synapses enable the LT-ST synaptic configuration to encode a better representation of the input patterns. We perform an iso-accuracy analysis to demonstrate the benefits of the proposed synaptic configuration. It can be observed from Fig. 11(b) that an SNN with 225 excitatory neurons and LT-ST synapses (352,800 synaptic units) provides a comparable classification performance to a network consisting of 400 excitatory neurons and one-bit synapses (313,600 synaptic units). The LT-ST synaptic configuration offers a 2× improvement in the programming energy consumption as illustrated in Fig. 11(c) due to faster training convergence while incurring a minimal area penalty. We note that the classification performance is strongly dependent on the parameters governing the stochastic STDP dynamics and the relative significance of the constituent synaptic elements, which needed to be tuned precisely to accomplish efficient synaptic learning at a faster rate of convergence.

### Power Comparison

We use a network comprising 225 excitatory neurons and LT-ST synapses to analyze the power consumption, since it provided the best trade-off among classification performance, area, and the number of training epochs. It can be seen from Fig. 11(c) that a total of 10.34 *μJ* was expended to program all the synapses for the entire training duration. The average programming energy is 29.3 *pJ* per synapse. The power consumption is subsequently estimated from the average programming energy and the total training period.


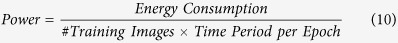


It is determined to be 182 *pW* per synapse for the network under consideration that was trained using 460 images from the MNIST dataset for a time period of 350 *μs* per epoch. We note that the neuromorphic system is simulated at an accelerated time scale, wherein the network parameters including the membrane potential and synaptic currents are updated every 0.5 *μs*. The proposed MTJ-HM based stochastic synapse is demonstrated to be power efficient in comparison to state-of-the-art CMOS synapses^24^,^27^.

## Conclusion

SNNs are a powerful neuromorphic computing paradigm that aim to mimic the computational efficiency of the human brain to solve complex inference tasks. However, custom SNN architectures implemented using standard CMOS technology have been shown to be power inefficient. In this work, we put forward a three terminal MTJ-HM heterostructure as a stochastic binary synapse. We presented the stochastic STDP learning algorithm to achieve plasticity in one-bit synapses, and demonstrated its efficacy on an SNN trained for handwritten digit recognition. Finally, we proposed a significance driven LT-ST stochastic synapse that is composed of two distinct binary synaptic elements, in order to further augment the efficiency of synaptic learning. This improved the classification performance of the SNN while achieving faster convergence during the training phase. Our iso-accuracy analysis shows that a neuromorphic system employing the LT-ST synapses offers a 2× improvement in the programming energy consumption over a network of one-bit synapses.

## Additional Information

**How to cite this article**: Srinivasan, G. *et al*. Magnetic Tunnel Junction Based Long-Term Short-Term Stochastic Synapse for a Spiking Neural Network with On-Chip STDP Learning. *Sci. Rep.* **6**, 29545; doi: 10.1038/srep29545 (2016).

## Figures and Tables

**Table 1:** MTJ-HM Device Simulation Parameters.

Parameters	Value
Free layer area	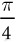 × 100 × 40 *nm*^2^
Free layer thickness	1.2 *nm*
Heavy metal thickness, *t*_*HM*_	2 *nm*
Saturation Magnetization, *M*_*s*_	1000 *KA/m*^16^
spin-Hall Angle, *θ*_*SH*_	0.3^16^
Gilbert damping factor, *α*	0.0122^16^
Energy Barrier, *E*_*B*_	20 *K_B_T*
Resistivity of HM, *ρ*_*HM*_	200 *μΩ.cm*^16^
Programming pulse width, *t*_*PW*_	1.0 *ns*
Temperature, *T*	300 *K*

**Table 2:** STDP Learning Circuit Parameters.

Parameters	Value
Supply Voltage, *V*_*DD*_	1 *V*
Reset Voltage, *V*_*RESET*_	320 *mV*
Bias Voltage, *V*_*T*_*POT*_ (One-Bit Synapse)	960 *mV*
Bias Voltage, *V*_*T*_*POT*_ (LT Synapse)	975 *mV*
Bias Voltage, *V*_*T*_*POT*_ (ST Synapse)	980 *mV*
Capacitance, *C*_*POT*_ (One-Bit Synapse)	500 *fF*
Capacitance, *C*_*POT*_ (LT Synapse)	500 *fF*
Capacitance, *C*_*POT*_ (ST Synapse)	3 *pF*
Heavy Metal Resistance, *R*_*HM*_	400 Ω
